# VDAC1 as Pharmacological Target in Cancer and Neurodegeneration: Focus on Its Role in Apoptosis

**DOI:** 10.3389/fchem.2018.00108

**Published:** 2018-04-06

**Authors:** Andrea Magrì, Simona Reina, Vito De Pinto

**Affiliations:** ^1^Section of Molecular Biology, Department of Biological, Geological and Environmental Sciences, University of Catania, Catania, Italy; ^2^Section of Biology and Genetics, Department of Biomedicine and Biotechnology, National Institute for Biomembranes and Biosystems, Section of Catania, Catania, Italy

**Keywords:** mitochondria, apoptosis, VDAC, peptides, oligos, microRNAs, biological drugs

## Abstract

Cancer and neurodegeneration are different classes of diseases that share the involvement of mitochondria in their pathogenesis. Whereas the high glycolytic rate (the so-called Warburg metabolism) and the suppression of apoptosis are key elements for the establishment and maintenance of cancer cells, mitochondrial dysfunction and increased cell death mark neurodegeneration. As a main actor in the regulation of cell metabolism and apoptosis, VDAC may represent the common point between these two broad families of pathologies. Located in the outer mitochondrial membrane, VDAC forms channels that control the flux of ions and metabolites across the mitochondrion thus mediating the organelle's cross-talk with the rest of the cell. Furthermore, the interaction with both pro-apoptotic and anti-apoptotic factors makes VDAC a gatekeeper for mitochondria-mediated cell death and survival signaling pathways. Unfortunately, the lack of an evident druggability of this protein, since it has no defined binding or active sites, makes the quest for VDAC interacting molecules a difficult tale. Pharmacologically active molecules of different classes have been proposed to hit cancer and neurodegeneration. In this work, we provide an exhaustive and detailed survey of all the molecules, peptides, and microRNAs that exploit VDAC in the treatment of the two examined classes of pathologies. The mechanism of action and the potential or effectiveness of each compound are discussed.

## Introduction

Mitochondria are crucial organelles for eukaryotic cells since they support the huge demand of energy required to maintain cellular homeostasis. Metabolites and ions are, thus, continuously exchanged with the cytosol through the Mitochondrial Outer Membrane (MOM), which owes its selective permeability mainly to the presence of mitochondrial porins, known as Voltage-Dependent Anion Channel (VDAC) (Shoshan-Barmatz et al., 2010). VDACs are the most abundant pore-forming proteins of the MOM and, differently from other structurally similar proteins such as Tom40 or Sam50, they serve as unspecific channels allowing the exchanges of molecules up to a molecular weight of 1,500 Da. High conserved through evolution, in mammals three distinct genes encode for three different VDAC isoforms, namely VDAC1, VDAC2, and VDAC3. The three isoforms are characterized by similar molecular weight of 28–32 kDa and by about 70% of sequence similarity (Sampson et al., 1997; Messina et al., 2012), all features suggesting a common tridimensional structure. Nevertheless, the three proteins display different roles in physiological and pathological conditions, as well as different expression level and tissue-specificity. Beyond the metabolic functions, the peculiar position of VDACs, at the interface between cytosol and mitochondria, makes porins the mitochondrial docking site for several cytosolic proteins, including molecules involved in the regulation of cell life and death. In this perspective, VDAC proteins appear as regulator of apoptosis, exerting both pro- and/or anti-apoptotic functions in physiological and pathological condition. Many pathologies, such as cancer and neurodegenerative disorders, indeed, show a deregulation of apoptosis pathways that correlates with alteration of VDAC activity, expression and functionality. For this reason, VDAC proteins have quickly become a new pharmacological target, and many molecules and peptides have been developed so far, aimed to modulate VDACs activity and ability to regulate apoptosis, with the final goal to find new therapeutic strategies for many disease treatments. In this review, we have grouped and described molecules and peptides with both pro-apoptotic and pro-survival properties. These molecules have been associated with different pathologies and while several of them are well known and already used in clinical trials, other new molecules, just assayed *in vitro* or at the cellular level, have been surveyed here.

## Structure and function of VDAC proteins

**Figure 1:**
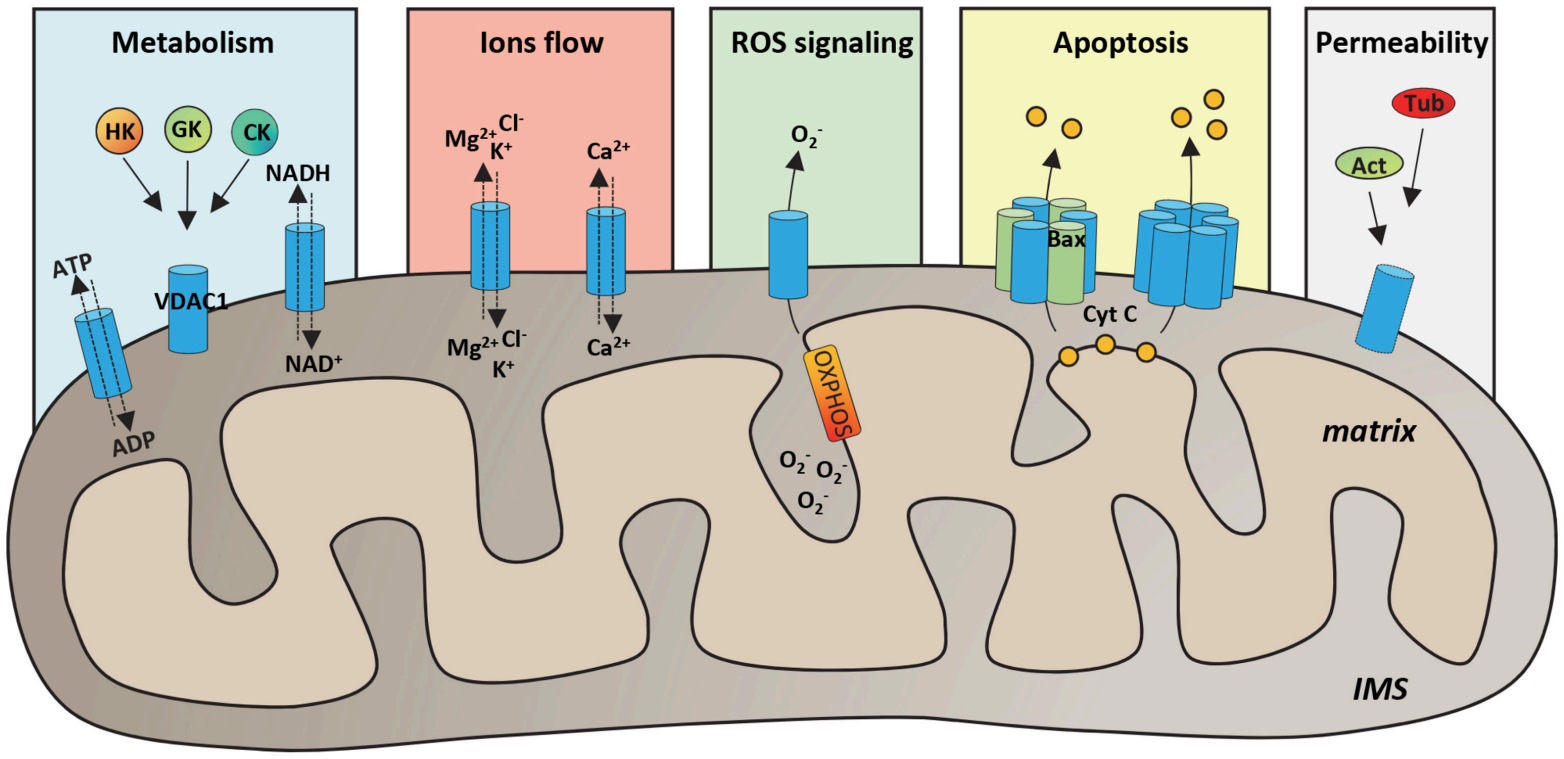
Functional roles of VDAC1 in physiological conditions. Schematic representation of VDAC1 functions in the cell. VDAC1 serves as the main gate in the MOM for metabolites, such as ATP/ADP and NAD^+^/NADH, but also Krebs cycle's intermediates, cholesterol and glutamate. Furthermore, by interaction with many cytosolic enzymes, such as Hexokinases (HK), Glucokinase (GK), and Creatine Kinase (CK), VDAC1 provides the ATP source essential for enzyme's activity. VDAC1 controls the flux of magnesium, chloride and potassium ions across the MOM, as well as of calcium, participating in the maintenance of cytosolic Ca^2+^ level in the physiological range. Evidence highlighted that VDAC1 acts as a preferential release channel for the hydrophilic ROS superoxide anion, produced during respiration by OXPHOS. Moreover, VDAC1 is considered a regulator of apoptosis; indeed, under apoptotic stimuli, VDAC1 undergoes oligomerization, by interacting with the pro-apoptotic protein Bax or with other VDAC1 molecules and constituting a channel big enough to promote cytochrome c (CYT C) releases to the cytosol and activation of apoptosis. It has been showed that many cytoskeleton proteins, such as Actin (Act) or Tubulin (Tub) bind VDAC1 participating in the regulation of channel permeability.

VDAC proteins are crucial for the metabolic cross talk between cytosol and mitochondria. Through VDACs, the newly synthetized ATP is continuously exchanged with ADP, as well as NAD^+^/NADH and many Krebs's cycle intermediates (Benz, 1994; Hodge and Colombini, 1997; Rostovtseva and Colombini, 1997; Lee et al., 1998). VDAC proteins regulate the flux of small ions (Cl^−^, K^+^, Na^+^, and Ca^2+^), participate in fatty acid transport across the MOM and in cholesterol distribution in mitochondrial membranes (Campbell and Chan, 2008; Lee et al., 2011). Furthermore, VDACs participate in the regulation of calcium concentration, maintaining the physiological level of cytosolic calcium, and are the channels responsible of ROS (superoxide anion) release to the cytosol (Han et al., 2003; Simamura et al., 2008a; De Stefani et al., 2012). Through the interaction with many metabolic enzymes, such as hexokinases, glycerol kinase (Fiek et al., 1982), glucokinase, and creatine kinase (Brdiczka et al., 1994), VDACs take part in the control of glycolytic metabolism. The main VDAC cellular functions are summarized in Figure 1.

**Figure 2:**
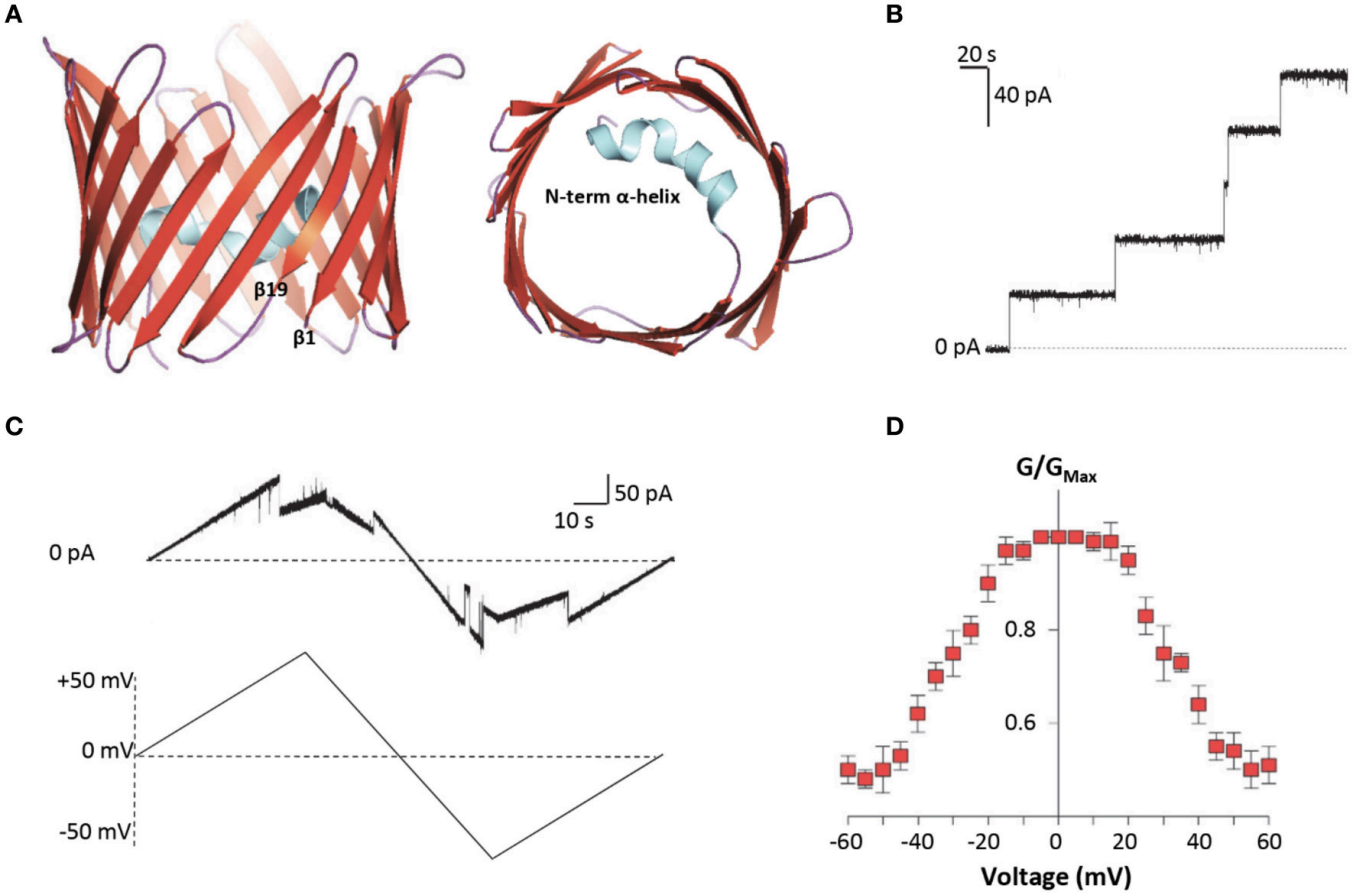
Structure and electrophysiological features of human VDAC1. **(A)** Three-dimensional structure of human VDAC1 from the side or top view. VDAC1 is a β-barrel (in red) formed by 19 anti-parallel β-strands, with the exclusion of β1 and β19 which are parallel. The strands are connected by loops (in purple). The N-terminal domain (in light blue) is arranged in α-helix and it is located inside the pore's lumen. This structure was drawn by PyMol software and is based on the hVDAC1 (PDB 5XDN). **(B)** Representative trace of recombinant hVDAC1 insertion in artificial membrane measured at the PLB. The trace indicates that hVDAC1 can easily form channels of about 4 nS in 1 M KCl. The experiment was performed at the constant voltage of + 10 mV. **(C)** Representative triangular curve of recombinant hVDAC1 showing changes in channel conductance upon application of a voltage ramp between ± 50 mV. As shown, hVDAC1 remains in a stable high-conductive state at low voltages, between ± 30 mV; conversely, at higher voltages, hVDAC1 switches into low-conductive states. The experiment was performed in 1 M KCl. **(D)** Bell-shaped curve of hVDAC1 voltage dependence, showing the channel's open probability (G/G_Max_) in relation to the voltage applied. Data are expressed as mean of G/G_Max_ ± SEM of *n* = 3 independent experiments, performed in 1 M KCl in a voltage range of ± 60 mV.

Among VDAC isoforms, VDAC1 is the most abundant and best characterized one. In 2008, the crystallographic structure of mouse VDAC1 was solved by means of X-ray diffraction and confirmed by NMR in the human protein. As reported in Figure 2A, VDAC1 is organized as a transmembrane β-barrel, made by 19 anti-parallel β-strands, while the N-terminal domain, including the first 25 amino acids, is structured in α-helix short stretch, and is localized inside the pore's lumen (Bayrhuber et al., 2008; Hiller et al., 2008; Ujwal et al., 2008). The N-terminal domain is suspected to participate in the stabilization of the pore's structure by its interaction with the channel's wall (Villinger et al., 2010) but, at the same time, it is considered the mobile part of the protein, being exposed to the cytosol under certain conditions (Geula et al., 2012; Tomasello et al., 2013). The 3D structures of the others human VDAC isoforms remain unsolved so far. Recently, VDAC2 from zebrafish was crystalized, showing to have a structure very similar to that of VDAC1 (Schredelseker et al., 2014). Due to the high sequence similarity between the three isoforms, homology modeling studies predict similar 3D structure for VDAC2 and VDAC3. An exception is the N-terminal domain of VDAC2, which is longer than the other two isoforms and cannot be modeled (De Pinto et al., 2010a). Furthermore, homology modeling revealed high similarity also with porins extracted from *Saccharomyces cerevisiae* (Guardiani et al., 2018): the ability of human and mouse VDAC isoforms to complement the lack of the endogenous porin1 in yeast (Reina et al., 2010; Magri et al., 2016a) confirmed that VDACs are made with a common motif.

Electrophysiological techniques are widely used to characterize the electrophysiological features of VDAC channels from many organisms (De Pinto et al., 1989; Palmieri and De Pinto, 1989; Aiello et al., 2004; Reina et al., 2013; Guardiani et al., 2018). Mammalian VDAC1 and VDAC2 easily open pores into an artificial membrane formed in a Planar Lipid Bilayer (PLB) apparatus: the pore-forming activity is studied in terms of conductance increase through the otherwise not conducting phospholipidic membrane (Figure 2B). Different VDAC proteins may show differences in their electrophysiological features, in dependence of the sequence of the protein, the phospholipids in the membrane (Brenner and Lemoine, 2014) and the applied voltage. E.g., high cholesterol can impair the activity of many membranes' proteins and, specifically, inhibit VDAC function (Campbell and Chan, 2008). In general, however, they have a typical behavior that can be easily recognized: the formation of the pores is discrete, with a stepwise appearance, and the known VDAC proteins are more or less uniform in this appearance (Benz et al., 1989; Menzel et al., 2009; Guardiani et al., 2018). VDACs are characterized by different conductance values accordingly to the applied voltage. This phenomenon is called voltage-dependence, and it is well known for the channels. *In vitro*, in reconstitution experiments where VDACs are inserted in an artificial membrane, when a low voltage between ± 20 mV is applied, both VDAC1 and VDAC2 display a high-conductive state (known also as “open” state), with conductance value of about 3.5–4.0 nS in 1 M KCl or NaCl (Colombini, 1980; Xu et al., 1999; Gattin et al., 2015). However, as the voltage increases (in positive or negative sign), VDAC1 and VDAC2 undergo several low-conducting (“closed”) states (see Figures 2C,D). These features are well conserved in the evolution for many VDAC isoforms: e.g., similar electrophysiological features were found for VDAC1 and, more recently, for VDAC2 from bovine spermatozoa (Menzel et al., 2009) and for VDAC2 extracted from yeast *S. cerevisiae* (Guardiani et al., 2018). On the contrary, human VDAC3 shows a very low propensity to form pores into PLB. VDAC3 channels are characterized by a very low conductance (about 100 pS in 1 M KCl) without showing any voltage-dependence (Checchetto et al., 2014; Okazaki et al., 2015). This peculiar behavior of VDAC3 possibly depends from the high oxidation level of cysteines (Reina et al., 2016a), a specific feature which suggests a putative role of VDAC3 in the redox signaling and mitochondrial quality control (De Pinto et al., 2016; Reina et al., 2016b). Accordingly, the analysis of VDAC3 interactome has highlighted the propensity of this isoform to bind redox enzyme and stress-sensor proteins (Messina et al., 2014), supporting this hypothesis.

## VDAC in apoptosis regulation

Mitochondria play a key role both in intrinsic and extrinsic pathways of apoptosis. Mitochondria contain a set of apoptogenic factors, including cytochrome c (cyt c), AIF and Smac/Diablo. In physiological conditions, apoptogenic factors are normally located in the intermembrane space of mitochondria (IMS). However, under apoptotic stimuli, they are released into the cytosol, leading to cyt c interaction with APAF-1 and the formation of apoptosome, which in turn activates the caspases cascade (Wang and Youle, 2009; Vaux, 2011). The release of cyt c to the cytosol occurs by the alteration of MOM permeability, a mechanism finely regulated by Bcl-2 proteins. Bcl-2 represents a heterogeneous family of both pro- and anti-apoptotic proteins, characterized by the presence of the common domain BH3. Bcl-2 proteins are mainly cytosolic; however, after certain stimuli, they translocate to the mitochondria, promoting the MOM permeabilization (Martinou and Youle, 2011). Many intrinsic stimuli, such as increased cytoplasmic level of Ca^2+^, severe oxidative stress, DNA damages and hypoxia (Le Bras et al., 2005; Keeble and Gilmore, 2007; Kroemer and Zitvogel, 2007), promote the mitochondrial translocation of the pro-apoptotic protein Bax and its interaction with the mitochondrial-located Bak, through conformational changes that leads to the formation of hetero-oligomers big enough to allow the passage of apoptogenic factors (Gross et al., 1998; Kroemer et al., 2007). Similarly, an extrinsic signal, e.g., the binding of an extracellular molecule to a specific receptor on the plasma membrane, results in the activation of caspase-8 that, in turn, promotes the cleavage of the pro-apoptotic protein Bid. The truncated Bid form, tBid, translocates into MOM, and interacts with Bak, participating in hetero-oligomers formation (Korsmeyer et al., 2000). In this contest, VDAC proteins participate in the regulation of mitochondrial-mediated apoptosis in different ways. In particular, while VDAC1 is widely considered a pro-apoptotic protein (see below), VDAC2 exerts an anti-apoptotic function. On the contrary, no information about the involvement of VDAC3 in apoptosis regulation is available so far.

Much evidence indeed indicates a specific function as pro-survival protein for VDAC2. This suggests a co-evolution of this mammalian-specific VDAC isoform with Bcl-2 proteins to regulate cell death (Cheng et al., 2003). In fact, VDAC2 specifically binds Bak, sequestrating it into the MOM in an inactive conformer and, thus, inhibiting Bak-dependent mitochondrial apoptosis (Cheng et al., 2003). Only recently, the specific domain of VDAC2 necessary for BAK interaction was identified (Naghdi et al., 2015). A similar mechanism of VDAC2-mediated inhibition was found also for the cytosolic protein Bax, which was partially found in the MOM and associated to VDAC2 (Ma et al., 2014).

Conversely, VDAC1 is able to bind Bax exerting a pro-apoptotic activity. In particular, the interaction of Bax with VDAC1 not only blocks the ATP/ADP exchange, affecting the channel functioning (Vander Heiden et al., 1999), but leads to the formation of hetero-oligomers VDAC1-Bax involved in cyt c release and caspase cascade activation (Shimizu et al., 1999; Shimizu and Tsujimoto, 2000). Alternatively, apoptotic stimuli are able to induce VDAC1 oligomerization, leading to the formation of channels big enough to allow the passage of cyt c to the cytosol (Keinan et al., 2010).

An early theory indicated that opening of the mitochondrial Permeability Transition Pore (mPTP), led to loss of the mitochondrial membrane potential, mitochondrial swelling, and the rupture of the MOM (Zoratti and Szabò, 1995; Halestrap et al., 1997). In an old model, mPTP was proposed to be formed by VDAC1 in the MOM, adenine nucleotide translocator (ANT) in the IMM, and cyclophilin-D (CyP-D) in the matrix (Marzo et al., 1998; Bernardi, 1999; Green and Evan, 2002; Tsujimoto and Shimizu, 2007). However, mPTP opening in VDAC1- or ANT-null cells (Baines et al., 2007), have challenged the mPTP model, which remains a not completely answered question.

Pro-apoptotic properties of VDAC1 are prevented by its interaction with the metabolic enzymes hexokinases (HKs). The two main HK isoforms, namely HK1 and HK2, are both involved in the first rate-limiting step of glycolysis. Both isoforms bind to VDAC1, obtaining a direct access to mitochondrial ATP, despite HK2 shows a higher affinity for mitochondrial binding (Wilson, 2003). The biological significance of VDAC1-HKs complexes is more profound. Indeed, HKs compete with Bax for binding to VDAC1, reducing the formation of VDAC1-Bax complexes (Vyssokikh et al., 2002); conversely, HKs detachment from VDAC1 induces apoptosis, increasing VDAC1 propensity to bind Bax or to participate in hetero- or homo-oligomeric structure formation (Abu-Hamad et al., 2008). Not coincidentally, VDAC1-HKs complexes are exploited in tumors, since mitochondrial HK2 increases the glycolysis rate, participating to the “Warburg effect,” and protects cancer cells from apoptosis (Gatenby and Gillies, 2004; Pedersen, 2008).

### VDAC-mediated apoptosis as a target for many disease's treatment

VDAC proteins play a crucial role in controlling mitochondrial metabolism and apoptosis. In this perspective, VDACs become interesting from a pharmacological point of view and many molecules targeted to VDAC proteins have been developed so far. Indeed, both alterations of apoptosis and of mitochondrial bioenergetics represent basal molecular mechanisms whose modulation is present in many pathologies.

A cancer hallmark is the apoptosis inhibition. A combination of factors leads to raising cell resistance to death stimuli. In many tumors, several anti-apoptotic proteins are overexpressed (Strasser et al., 1990; Adams and Cory, 2007) and the rapid growth of malignant cells is strongly supported by VDAC1-HKs complexes, which increase glucose metabolism and inhibit apoptosis (Mathupala et al., 2006). Conversely, the detachment of HKs from VDAC1 promotes the channel propensity to interact with the pro-apoptotic Bax and Bak proteins (Majewski et al., 2004) or to form VDAC1 oligomers (Keinan et al., 2010). Therefore, therapeutic approaches aimed to counteract malignant cells proliferation have taken into account strategies directed to induce HKs detachment from VDAC1 and/or to act on VDAC1 expression and channel activity.

In cancer, cell death is significantly inhibited, but in neurodegenerative disease, on the opposite, the early onset of neuron's death is among the causes of the pathologies. Neurodegenerative disorders represent a large group of age-related pathologies, which affect different nervous system's regions. Among disorders affecting brain, the most studied are undoubtedly Alzheimer's disease (AD) and Parkinson's disease (PD), while the most known neuromuscular disorder is Amyotrophic Lateral Sclerosis (ALS), which specifically affects spinal cord. Pathologies such as AD, PD, and ALS are characterized by different etiologies and symptoms. Nevertheless, at the molecular level, they are characterized by accumulation within the cells of misfolded proteins and/or peptide which can directly interact with VDAC1 (Magrì and Messina, 2017). The interaction of misfolded protein with VDAC1 has dramatic consequences for mitochondrial functionality. At the same time, in AD and in PD, a significative alteration of caspase-mediated apoptotic pathways was found (Li et al., 2000; Rohn et al., 2009), which correlated with a reduction of mitochondrial rate of HKs (Israelson et al., 2010; Smilansky et al., 2015; Magrì et al., 2016b). Furthermore, the analysis of post-mortem brain from AD patients and transgenic mice have shown that VDAC1 is over-expressed and that the level of VDAC1 phosphorylation is significantly increased (Cuadrado-Tejedor et al., 2011). In this perspective, molecules able to interfere with misfolded proteins interaction with VDAC1 and/or decrease the pro-apoptotic features due to the overexpression of the channel have been proposed as therapeutic tool. In this review, we grouped molecules that, by acting on VDAC1, exert pro- and anti-apoptotic features, thus putatively able to counteract mitochondrial dysfunction in cancer and neurodegenerative diseases, respectively. However, the targeted delivery of drugs to specific intracellular locations is one of the most challenging obstacle to be overcome. Several chemotherapeutic drug cannot easily cross the protective, physiological barriers in tumor tissues. For this reason, we paid specific attention onto biological molecules like peptides and oligos, which definitely represent a most promising alternative to conventional molecules used nowadays against cancer and neurodegeneration.

## VDAC targeted molecules that affect apoptosis

### Pro-apototic molecules acting on VDAC1 channel activity

**Figure 3:**
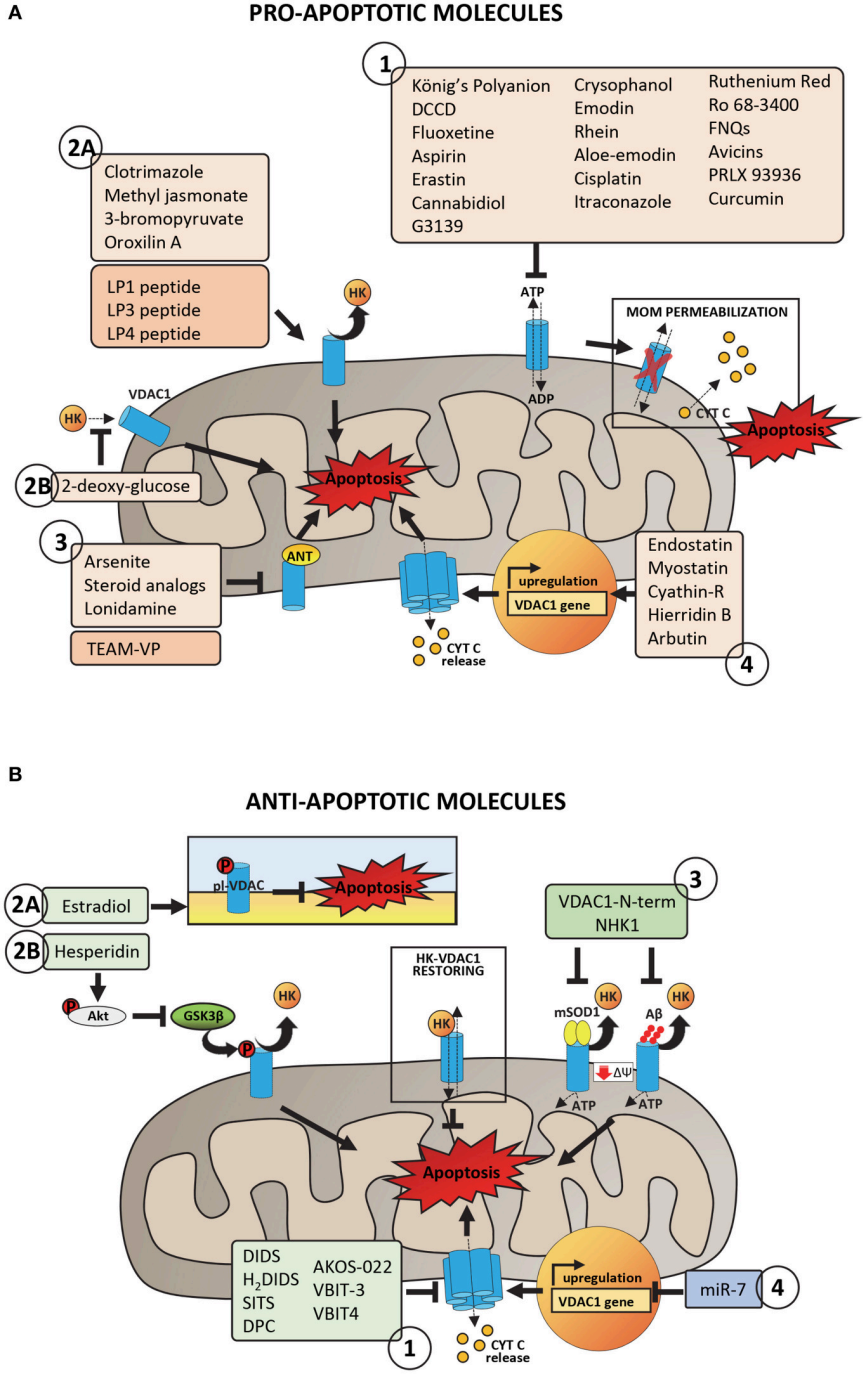
Pro- and anti-apoptotic molecules acting on VDAC1 and putatively involved in pharmacological treatment of cancer and neurodegeneration. **(A)** Pro-apoptotic molecules and peptides acting on VDAC1 with proven or potential role in the pharmacological treatment of cancer phenotype. Group 1 includes molecules acting on VDAC1 channel activity by promoting the impairment of metabolic exchanges between mitochondria and cytosol, leading to MOM permeabilization and activation of apoptosis. Group 2 includes molecules and peptides acting on VDAC1-HKs complexes by promoting HKs detachment from VDAC1 (2A) or preventing HKs binding to VDAC1 (2B). Group 3 includes molecules and peptides acting on ANT-VDAC1 complexes (the precise mechanism is still unclear). Group 4 includes molecules inducing VDAC1 overexpression and the consequent propensity of VDAC1 to form oligomers. **(B)** Anti-apoptotic molecules and peptides acting on VDAC1 and potentially able to reduce mitochondrial dysfunction in neurodegenerative diseases. Group 1 includes peptides with proven ability to bind VDAC1 and to impair the aggregation of misfolded SOD1 mutants or Aβ peptide with VDAC1, restoring VDAC1-HKs complexes, and VDAC1 functionality. Group 2 includes molecules acting on VDAC1 phosphorylation at both plasma membrane (2A) and mitochondrial (2B) level with consequences on VDAC1 channel activity or the ability to bind HKs. Group 3 includes channel blockers, molecules with proven ability to bind specifically VDAC1 and to counteract the VDAC1 oligomerization. Group 4 includes siRNA able to downregulate VDAC1 expression, decreasing in turn the VDAC1 propensity to form oligomers.

Over the years, various anti-cancer molecules able to directly target to VDAC1 were proposed (Reina and De Pinto, 2017). Most of described molecules directly interact with VDAC1, reducing the channel conductance, eventually leading to apoptosis (Figure 3A, Group 1). The **König's Polyanion (KPa)**, a 1:2:3 copolymer of methacrylate, maleate, and styrene, is surely one of the first listed compound able to lower VDAC's gating voltage and induce irreversible channel closure *in vitro* (König et al., 1982; Colombini et al., 1987; Tedeschi et al., 1987; Benz et al., 1988; Mannella and Guo, 1990). Nevertheless, the existence of contrasting results that prove both apoptotic and anti-apoptotic effects together with the lack of specificity for VDAC, prevent KPa utilization as an anticancer drug. Likewise, **dicyclohexylcarbodiimide (DCCD)** has been reported to inhibit hexokinase binding by covalently labeling VDAC (Nakashima et al., 1986; Nakashima, 1989; De Pinto et al., 1993; Shafir et al., 1998) and blocking its channel activity (Shafir et al., 1998). The high specificity of this interaction is strengthened by the identification of VDAC-Glu72 amino residue as the binding site of both DCCD and hexokinase (De Pinto et al., 1993; Zaid et al., 2005). In spite of this, the ability of DCCD to inhibit various ATPases prohibits its use in humans.

Several molecules have been intensively tested *in cellulo*. For instance, **fluoxetine**, a drug prescribed for the treatment of major depressive disorders, has been reported to inhibit proliferation of several cancer cells lines (Serafeim et al., 2003; Krishnan et al., 2008; Stepulak et al., 2008; Lee et al., 2010; Mun et al., 2013), although previous studies have associated the administration of this compound to an increased risk of developing tumor (Brandes et al., 1992; Lee et al., 2001). Once penetrated inside the cell, fluoxetine binds mainly to mitochondria (Mukherjee et al., 1998), most likely using VDAC, as demonstrated by PLB assays. In this conditions indeed, fluoxetine interacts with VDAC, altering its channel properties (Nahon et al., 2005; Thinnes, 2005). A recent report, however, questions the specificity of fluoxetine for VDAC, proposing its interaction with the Glutamate receptor 1 (GluR1) to trigger apoptosis in glioma cells (Liu et al., 2015). **Aspirin**, the famous nonsteroidal anti-inflammatory drug used as an antipyretic and analgesic agent, has been lately associated with a pro-apoptotic activity against different cancer cell types, such as colon cancer, chronic lymphocytic leukemia and myeloid leukemia. Tewari et al. demonstrated a direct modulation of membrane-reconstituted VDAC1 by aspirin, suggesting this interaction as responsible for the anticancer effects of the drug (Tewari et al., 2017). Following VDAC1 binding, indeed, aspirin would dissipate mitochondrial membrane potential (Δψm), dissociate HK2 from mitochondria and promote cell death. Accordingly, silencing of VDAC1 protects HeLa cells from aspirin-induced cell death. **Erastin** is another anti-tumor agent that use VDAC as a docking site in mitochondria and induces oxidative, non-apoptotic death in human tumor cells with mutations in the oncogenes HRAS, KRAS, or BRAF. Erastin specifically binds VDAC isoforms 2 and 3 (Yagoda et al., 2007), as confirmed by knockdown experiments, and it also modulates VDAC-tubulin interaction in proliferating cells (Maldonado et al., 2013). VDAC has been also proposed as a target for **cannabidiol (CBD)**, a phytocannabinoid derived from *Cannabis* species and devoid of psychoactive activity. Reconstitution experiments in artificial membranes demonstrated indeed the ability of CBD to directly bind VDAC, markedly decreasing its channel conductance. This interaction, further confirmed by microscale thermophoresis analysis (Rimmerman et al., 2013), may be responsible for the strong anti-tumor effects of cannabidiol observed in numerous cancer cells types (Ligresti et al., 2006; Massi et al., 2013). **Avicins** represent a class of natural compounds reported to target and close VDAC in lipid bilayers, causing the OMM permeabilization and the release of cytochrome *c* (Lemeshko et al., 2006; Haridas et al., 2007). Once again, such a mechanism would explain the pro-apoptotic effect of these triterpenoid saponines in tumor cells (Haridas et al., 2001; Mujoo et al., 2001; Gaikwad et al., 2005). Interestingly, avicins can still trigger cell death via autophagy even when Bax or Bak genes are deleted or caspases are inhibited, suggesting potential therapeutic activity in apoptosis-resistant cancers. Recently, **chrysophanol, emodin, rhein, aloe-emodin**, and **catechin**, the bioactive anti-cancer components of the herb *Rheum officinale Baill*, have been reported to bind VDAC through Thr207 and the N-terminal region of the protein (Li et al., 2017). A survey of literatures shows that these derivatives induce apoptosis in many human cancer cell lines, including lung adenocarcinoma A549, cervical carcinoma HeLa, and hepatoma HepG2 cells (Chu et al., 2012). Given the importance of VDAC as an anti-cancer target, the authors proposed a key role for this interaction in the cytotoxic activity of the compounds. Electrophysiological experiments have demonstrated the ability of **ruthenium Red (RuR)**, a water-soluble *hexavalent* polycation whose effects on apoptosis are debated (Anghileri, 1975; Zaid et al., 2005), to induce channel closure of membrane-reconstituted VDAC1 as well (Israelson et al., 2008). Interestingly, RuR would interact with the same VDAC1 loops responsible for HK1 binding (Israelson et al., 2008). Although with a slightly different mechanism, it is worth mentioning also **furanonaphtoquinones (FNQs)**, a class of highly reactive molecules that induce caspase-dependent apoptosis via ROS production. VDAC was proposed as the pharmacological target of **FNQs**, since their anti-cancer activity was increased upon VDAC1 overexpression and decreased upon VDAC1 silencing by siRNA (Simamura et al., 2006, 2008b). In 2003, Cesura et al. identified VDAC1 as the major molecular target of the PTP inhibitor **Ro 68-3400** in mitochondria prepared from a human neuroblastoma cell line. Later, however, this statement underwent several confutations (Cesura et al., 2003). Initially, single channel analysis revealed that Ro 68-3400 failed to alter the electrophysiological properties of VDAC1 incorporated into lipid membranes and afterwards, Bernardi and coworkers proposed the Mitochondrial Phosphate Carrier (PiC) as the proper interactor (Krauskopf et al., 2006).

Compounds that are already part of clinical trials conclude this list. Among them, **cisplatin** is one of the best known chemotherapeutic drug for the treatment of numerous human cancers (Khan et al., 1982; Scher and Norton, 1992; Abrams et al., 2003; Koch et al., 2013). Several clues about the enhanced sensitivity to cisplatin of cells with increased expression of VDAC1, suggested that this protein may serve as a cisplatin receptor in the apoptotic pathway (Thinnes, 2009). According to Keinan et al. cisplatin would induce cell death through VDAC1 oligomerization (Keinan et al., 2010), albeit discordant data proposed a significantly increased cytotoxicity of this drug in cancer cells silenced for VDAC1 (Wu et al., 2016). Clinical studies performed with **itraconazole**, a common antifungal drug, have demonstrated its potent antiangiogenic and anticancer activity. Again, VDAC would be part of the mechanism of action of this compound. Liu and coworkers, indeed, reported that the binding of itraconazole to VDAC1 causes an increase in the AMP:ATP ratio, which in turn activates AMPK that down-regulates mTOR pathway and thus inhibits cell proliferation (Head et al., 2015). VDAC has been recently proposed as the therapeutic target of **curcumin** as well. Curcumin (diferuloylmethane) is a component of the golden spice turmeric (*Curcuma longa)* with anti-inflammatory and antitumor activity (Aggarwal et al., 2003). Along with reports that claim curcumin capable of binding to Bcl2 proteins (Carroll et al., 2011; Rao et al., 2011; Yang et al., 2015), Tewari et al. firstly demonstrated its interaction with membrane-reconstituted VDAC1 (Tewari et al., 2015). Noteworthy, curcumin has already entered phase II clinical trial for the treatment of advanced pancreatic cancer (Dhillon et al., 2008) and the prevention of colorectal neoplasia (Carroll et al., 2011). Finally, a structural analog of the above mentioned erastin, called **PRLX 93936**, is currently in Phase I/II clinical trial for the treatment of patients with multiple myeloma (*ClinicalTrials.gov* database, NCI). As for erastin, it inhibits VDAC2 and VDAC3 in cells harboring mutations in the oncogenes HRAS, KRAS, and BRAF.

### Pro-apoptotic molecules counteracting the interaction of VDAC1 with hexokinases or the adenine nucleotide transporter

VDAC-hexokinase interaction certainly represents a crucial point in the establishment and maintenance of the cancerous metabolism. For this reason, one of the main classes of anti-cancer drugs targeted to VDAC specifically aims at destroying this bond (Figure 3A, Group 2). **Clotrimazole**, an antimycotic drug used in the treatment of fungal infections, is one of the best known molecule able to inhibit glycolysis by inducing the detachment of mitochondrial-bound hexokinase and therefore triggering apoptosis in various mouse models of cancer (Penso and Beitner, 1998; Snajdrova et al., 1998; Palchaudhuri et al., 2008; Kadavakollu et al., 2014). Unfortunately, despite many attempts to increase its bioavailability (Abdel-Moety et al., 2002; Prabagar et al., 2007; Yong et al., 2007), clotrimazole still has limited success in clinical use because of its poor solubility in water. Numerous reports also describe **3-bromopyruvate (3BrPA)** and **methyl jasmonate** as drugs involved in tumor suppression through detachment of hexokinase from VDAC (Galluzzi et al., 2008, 2013; Goldin et al., 2008; Cohen and Flescher, 2009; Cardaci et al., 2012; Ko et al., 2012; Pedersen, 2012; Shoshan, 2012). The first one is a pyruvate analog whose binding probably expose sites previously occupied by HK2 making them available to pro-apoptotic molecules, thus promoting the release of cytochrome *c* from mitochondria (Chen et al., 2009; Nakano et al., 2012). Several *in vitro* studies confirmed the extremely high selectivity of 3BrPA for malignant cells (Pedersen, 2007; Nakano et al., 2011). For many reasons, that we have not the space to discuss here, it has not yet entered in formal clinical trials, albeit human administration of 3BrPA has been reported (Ko et al., 2012; El Sayed et al., 2014). Methyl jasmonate is instead a plant stress hormone of the jasmonate family that showed to be highly selective toward cancer cells and ineffective toward normal cells (Fingrut and Flescher, 2002) and to have the ability to act against drug resistant cells (Fingrut et al., 2005). Although with a somewhat different mechanism, it is also worth mentioning in this context the **2-deoxy glucose (2DG)** that, inhibiting the activity of HK2, indirectly prevents its binding to VDAC. According to Ben Sahra et al., treatment with 2DG would promote cancer cell apoptosis when used in combination with the anti-diabetic drug metformin (Ben Sahra et al., 2010). Currently, 2-deoxy glucose is in phase I/II trial for the treatment of advanced cancer and hormone refractory prostate cancer (ClinicalTrials.gov database, NIH). A potent anti-tumor activity has been as well described for **oroxilin A**, an O-methylated flavone found in *Scutellaria baicalensis* and *Oroxylum indicum*. Besides several studies demonstrating its ability to induce apoptosis (Hu et al., 2006; Li et al., 2009; Zhao et al., 2010), to arrest cell cycle (Yang et al., 2008) and suppress metastasis in many cancer cell types, it was reported that oroxylin A induces dissociation of HK2 from mitochoia in human breast carcinoma cell lines (Wei et al., 2013). Despite conflicting opinions about the essential role for VDAC and adenine nucleotide translocase (ANT) in permeability transition pore (PTP), a channel whose opening leads to mitochondrial depolarization, VDAC-ANT complex is still recognized as an anti-cancer target (Beutner et al., 1998; Neuzil et al., 2013; Figure 3A, Group 3). Compounds that act at that level are **lonidamine**, **arsenites**, and **steroid analogs** (Belzacq et al., 2001). Interestingly, the arsenite analog **4-(N-(S-glutathionylacetyl)amino) phenylarsenoxide (GSAO)** was shown to inhibit ANT and to selectively kill proliferating angiogenic endothelial cells, while being non-toxic to growth-arrested endothelial cells (Don et al., 2003).

### Pro-apoptotic molecules controlling VDAC1 expression level

This third class of drugs includes few molecules. They act through less clear mechanisms than those described above, but all culminate in the modulation of VDAC expression levels (Figure 3A, Group 4). **Endostatin** is an example. The C-terminal globular domain of collagen XVIII is indeed a potent inhibitor of angiogenesis that promotes apoptosis by up-regulating VDAC1 expression. More specifically, this molecule seems to reduce HK2 expression, which, in turn, would lead to VDAC phosphorylation and accumulation (Yuan et al., 2008). **Myostatin**, a myokine of the transforming growth factor-β (TGF-β) superfamily, influences the expression levels of both HK2 and VDAC as well. Liu et al. proposed impaired VDAC and HK2 expression levels as responsible for HK2 dissociation from VDAC (Liu et al., 2013) and, consequently, for apoptosis induction in cancer cells. As reported in (Huang et al., 2015), **Cyathin-R** attenuates tumor growth and triggers apoptosis in Bax/Bak-deficient cells by modulating VDAC1 expression. This fungal-derived diterpenoid increases VDAC1 protein levels, thus supporting oligomerization that results in cell death. The involvement of VDAC is confirmed by the evidence that its silencing or inhibition of channel conductance and oligomerization completely abrogate Cyathin-R effects. Very recently, the marine metabolite **hierridin B** from *Cyanobium* sp. was proved to induce cytotoxicity selectively in HT-29 adenocarcinoma cells (Leão et al., 2013) through significant changes in VDAC1 mRNA expression and protein content (Freitas et al., 2016). From scarce information available, **arbutin**, a glycosylated hydroquinone extracted from the bearberry plant in the genus *Arctostaphylos* and widely used in cosmetics for its depigmenting effects would induce apoptosis in A375 human malignant melanoma cells by up-regulating VDAC1 (Nawarak et al., 2009).

### Anti-apoptotic molecules impairing VDAC1 oligomerization

Alzheimer's disease is characterized by an enanched expression levels of VDAC1 (Yoo et al., 2001; Cuadrado-Tejedor et al., 2011) and by reduced interaction of VDAC1 with the glycolityc enzymes HKs (Smilansky et al., 2015). These conditions increase VDAC1 propensity to form oligomers (Smilansky et al., 2015), promoting in turn the enhancement of apoptosis, with dramatic consequences for the early neuron's death (Mattson, 2000). Therefore, feasible therapeutic strategies to prevent apoptosis in AD include the downregulation of VDAC1 expression and/or the inhibition of VDAC1 oligomerization (Figure 3B, Group 1).

The 4,4′-diisothiocyanostilbene-2,2′-disulfonic acid, known as **DIDS**, is a calcium and chloride channel blocker (Cabantchik et al., 1978) which, when added to PLB-reconstituted VDAC1, is able to decrease the channel conductance (Thinnes et al., 1994). It has been demonstrated that DIDS exerts a pro-survival activity in HeLa cells treated with apoptotic inducers by preventing the activation of caspase-3, nuclear DNA fragmentation and cell volume decrease, common hallmarks of apoptosis activation (Benítez-Rangel et al., 2015). Although a direct inhibition of caspase's activity was recently proposed (Benítez-Rangel et al., 2015), DIDS is able to counteract apoptosis at the early stage, by diminishing VDAC1 oligomerization and thus preventing cyt c release to the cytosol (Keinan et al., 2010). In fact, treatment of HeLa cells with DIDS was able to counteract the toxicity of staurosporine, an established apoptotic inducer, via VDAC1 oligomerization (Keinan et al., 2010). Very similar results were achieved by using DIDS analogs (**H**_2_**DIDS, SITS, DPC**) which successfully counteracted cisplatin- or selenite-induced apoptosis in SH-SY5Y cells (a cell line commonly used as model of neurodegenerative diseases), again by diminishing the oligomerization of VDAC1 (Ben-Hail and Shoshan-Barmatz, 2016). The molecular mechanism correlating the channel block with VDAC1 oligomerization is not completely understood, even though several hypothesis have been proposed. For instance, DIDS binding to VDAC1 could interfere with Bax interaction with VDAC1 and, thus, formation of oligomers (Liu et al., 2008; Tajeddine et al., 2008).

A high-throughput compound screening approach led recently to the identification of other blockers exerting high specificity for VDAC1. One of them, called **AKOS-022**, was able to reduce significantly VDAC1 conductance at the PLB and decrease VDAC1 oligomerization already at micromolar concentration (Ben-Hail et al., 2016). Molecules based on the chemical structure of AKOS-022 were then synthetized, in order to maximize the anti-apoptotic effect. Among them, molecules named **VBIT-3** and **VIBT-4** have shown a pro-survival activity counteracting VDAC1 oligomerization in a pharmacological range of concentration and only in presence of VDAC1 overexpression, suggesting the pharmacological employment in AD (Ben-Hail et al., 2016).

### Anti-apoptotic molecules modulating post-translational modification of VDAC1

VDAC1 is subject of post-translational modifications, such as phosphorylation, oxidation, and acetylation (Kerner et al., 2012; Figure 3B, Group 2). In particular, phosphorylation occurs in specific serine, tyrosine or threonine residues and leads to the modulation of the channel activity and the regulation of apoptosis (Bera et al., 1995; Banerjee and Gosh, 2006). Many kinases have been found involved in VDAC1 modification, e.g., the glycogen synthase kinase 3 beta (GSK3β) phosphorylates VDAC1 on threonine 51 (Martel et al., 2013). The phosphorylation of VDAC1 Thr 51 exerts a strong effect on the channel ability to bind HKs: as the phosphorylation extent increases, indeed, the affinity of the glycolytic enzyme for VDAC1 diminishes (Martel et al., 2013). Therefore, activation of GSK3β favors apoptosis by promoting HKs detachment from VDAC1. In AD, the activity of GSK3β is significantly enhanced, resulting in a cascade events which include VDAC1 phosphorylation, detachment of mitochondrial HK1 and activation of apoptosis (Martel et al., 2013). At the same time, the enhanced activity of GSK3β correlated with the accumulation of Aβ peptide and the phosphorylation of Tau (Jope and Johnson, 2004; Jope et al., 2007). **Hesperidin** is a flavonoid found in *citrus* with known anti-inflammatory and anti-oxidant properties. The employment of hesperidin in AD has shown to be protective in different pathological models: e.g., in rat, the flavonoid was able to significantly improve the cognitive impairments typical of the pathology, by decreasing both oxidative stress and apoptosis rate (Justin Thenmozhi et al., 2017). The protective effect of hesperidin in AD is due to the modulation of Akt/GSK-3β pathway, a cascade mechanism that involves VDAC1. Hesperidin, indeed, promotes the phosphorylation of Akt, which once activated, reduces GSK-3β activity (Wang et al., 2013). As consequence, the phosphorylation rate of VDAC1 is significantly reduced while HK1 binding to VDAC1 is improved, supporting cell metabolism and cell growth (Wang et al., 2013).

An analog mechanism was found also for the VDAC1 portion localizing on the plasma membrane (pl-VDAC) (Thinnes et al., 1989). pl-VDAC is particularly abundant in hippocampus and frontal cortex and localizes in specialized membrane regions called lipid rafts (Bàthori et al., 1999; De Pinto et al., 2010b), where together with caveolin-1 and the estrogen receptor α-like (mER), pl-VDAC forms a large protein complex (Ramirez et al., 2009; Herrera et al., 2011). As for the mitochondrial counterpart, pl-VDAC takes part in the control of the extrinsic pathway of apoptosis, by controlling ions transport across the membrane (Akanda et al., 2008; Thinnes, 2010). It has been proposed a key role of pl-VDAC in mediating Aβ-toxicity (Marin et al., 2007; Ramirez et al., 2009). Not coincidentally, pl-VDAC is expressed in brain regions with cognitive functions and thus more susceptible to AD. Experimental evidences have highlighted a protective effect of the estrogen **estradiol** against Aβ-toxicity in different AD models (Sherwin and Henry, 2008; Correia et al., 2010). Estradiol, indeed, modulates the phosphorylation level of pl-VDAC and its channel activity: when phosphorylated by estradiol, pl-VDAC is maintained in a closed and inactive form, which protects cells from apoptosis activation (Herrera et al., 2011). Conversely, the de-phosphorylation of pl-VDAC, operated by the antiestrogen tamoxifen, promotes channel opening, a mechanism which correlates directly with apoptosis (Herrera et al., 2011).

## Biological molecules affecting VDAC functions

### Pro-apoptotic peptides interfering with VDAC1-HKs interaction

Anti-cancer peptides have primarily been designed to interfere with VDAC binding to some of its major interactors, such as hexokinase. These “interfering” peptides simply mimic VDAC or HK sequences strongly suspected to be involved in the protein-protein interaction: the rationale is to restrict the side effects generally associated to the utilization of chemical drugs. The mechanistic hypothesis behind the pro-apoptotic effects of peptides implies the establishment of a competition between them and the two interacting proteins (Figure 3A, Group 2). The first work to lead the way of using anti-tumor peptides was the paper by Arzoine and colleagues. In this report, synthetic peptides mimicking the N-terminal region and two cytoplasmic loops of VDAC, respectively **LP1**, **LP3**, and **LP4**, were found able to detach and even prevent hexokinase binding to VDAC (Arzoine et al., 2009). Subsequently, LP1 and LP4 peptides were further engineered by adding the *Antennapedia* homeodomain (HD*Antp*) from *Drosophila*, in order to increase their intracellular delivery. Interestingly, modified VDAC1-based peptides were proved to reduce the anti-apoptotic effects of Bcl-2 or Bcl-xL (Arbel and Shoshan-Barmatz, 2010; Arbel et al., 2012) and selectively kill Chronic Lymphocytic Leukemia cells (Prezma et al., 2013). Peptides designed on the first 15 amino acid residues of HK1 have been shown to induce apoptosis in different cancer cell types as well (Gelb et al., 1992). Beside those based on VDAC and HK1 sequences, also a peptide from *Lactobacillus casei* peptidoglycan has been reported to exert antitumor activity by detaching mitochondrial-bound hexokinase (Fichera et al., 2016). There are only few information regarding the existence of anticancer peptides able to directly inhibit VDAC activity. An example is **Mastoparan**, a peptide contained in wasp venom and initially considered capable of triggering apoptosis via VDAC binding (Shol'-ts et al., 1984; Pfeiffer et al., 1995). Recently, however, the pro-apoptotic effect of mastoparan has been associated to its ability to interact with the phospholipid phase of the membrane (Yamamoto et al., 2014). The structural analog, the highly cytotoxic **Mitoparan** (MitP) targets mitochondria and induces apoptosis in human cancer cells, with a mechanism in which the involvement of VDAC was not completely demonstrated (Jones et al., 2008). A single report makes the chimeric **TEAM-VP peptide**, composed of a short sequence from HIV-1 Vpr (Viral protein R) fused with a cyclic RGD motif, a member of this class. According to Borgne-Sanchez et al. (2007) indeed, TEAM-VP would induce apoptosis in endothelial cells by binding both ANT and VDAC.

### Pro-survival peptides contrasting interaction of misfolded protein with VDAC1

A common feature of neurodegenerative diseases is represented by the accumulation of misfolded protein and peptides upon the cytosolic surface of mitochondria or to the VDAC1. Both Aβ peptide and hyper-phosphorylated Tau co-immuno-precipitated with VDAC1 in AD patients and in 3xTg-AD mice (Manczak and Reddy, 2012). Similarly, αSyn was found co-precipitated with VDAC1 in *substantia nigra* of a rat model of PD (Lu et al., 2013). In the neuromuscular disorder ALS, several mutants, but not wild-type, SOD1 were found co-precipitated with VDAC1 exclusively in spinal cord's mitochondria (Israelson et al., 2010). The addition of misfolded proteins to the PLB-reconstituted VDAC1 resulted in a strong inhibition of channel conductance (Israelson et al., 2010; Magrì et al., 2016b), suggesting an impairment of metabolite exchanges through VDAC1. At the same time, as the misfolded proteins interact with VDAC1, the amount of HKs on the mitochondrial surface decreases (Smilansky et al., 2015; Magrì et al., 2016b), possibly altering apoptosis. In this contest, synthetic peptides mimicking specific protein domains can represent a promising therapeutic strategy: they are aimed to bind the bait proteins (for example VDAC1 itself), clogging the docking site normally available for the interaction with other protein(s) (Figure 3B, Group 3).

A first strategy consisted in the development of VDAC1-based peptides aimed to bind misfolded proteins. The N-terminal domain of VDAC1, including the first 26 amino acid residues, is commonly considered the mobile part and an exposed moiety of the protein, putatively involved in the binding of cytosolic proteins in physiological conditions (Shi et al., 2003; Geula et al., 2012), as well as of Aβ peptide in AD (Thinnes, 2011). Therefore, a peptide based on the first 26 amino-terminal residues of VDAC1, named VDAC1-N-Term peptide, was recently developed (Smilansky et al., 2015). By exploiting several techniques, such as Surface Plasmon Resonance, the ability of VDAC1-N-Term to bind Aβ peptide was confirmed (Smilansky et al., 2015). Moreover, treatment of PC12 cells with VDAC1-N-Term peptide, in presence of external Aβ, significantly reduces Aβ uptake within the cells as well as the Aβ-induced apoptosis (Smilansky et al., 2015), suggesting a protective role exerted by the peptide against Aβ. As for the mitochondrial VDAC1, the pl-VDAC is suspected to mediate the extracellular Aβ toxicity, possibly promoting the peptide internalization (Thinnes, 2011): in fact, SHSY5Y cells overexpressing pl-VDAC are much sensitive to Aβ peptide toxicity (Smilansky et al., 2015). Therefore, VDAC1-N-Term peptide decreases Aβ internalization and toxicity by binding Aβ, preventing thus its intracellular accumulation (Smilansky et al., 2015).

A second strategy consisted in the development of peptides able to bind VDAC1 and based onto the most known VDAC1 interacting proteins, such as HK1. This strategy was successfully applied in an ALS model, which is characterized by lower HKs expression in affected tissue (Magrì et al., 2016b; Magrì and Messina, 2017). In particular, a small peptide, corresponding to the sequence 2–12 of N-terminal domain of HK1, was developed, since this region is commonly recognized as important for the interaction with VDAC1. Again, *in vitro* techniques were used to prove the interaction of this peptide, named NHK1, with VDAC1 (Magrì et al., 2016b). Furthermore, by binding VDAC1, NHK1 impairs the interaction of SOD1 G93A with the channel as well as accumulation of SOD1 mutant on the cytosolic surface of MOM (Magrì et al., 2016b). Moreover, if expressed in NSC34 cells (a commonly recognized ALS cell line model), NHK1 is able to impair toxicity mediated by SOD1 G93A overexpression (Magrì et al., 2016b). Overall, the results suggest that NHK1 binds VDAC1 and counteracts the binding of SOD1 G93A with a very simple mechanism (Magrì et al., 2016b). Although no experimental evidence are available so far, it is however possible to hypothesize a similar application also in other pathological model, such as PD or AD, in which NHK1 could reduce the affinity of αSyn or Aβ for VDAC1.

### Oligonucleotides and microRNAs with pro- or anti-apoptotic features

A pharmacological molecule that appears to exploit VDAC as a mitochondrial target is **G3139**, an 18-mer phosphorothioate anti-sense oligonucleotide complementary to the first six codons of Bcl-2 mRNA. This drug has entered phase III clinical trials in different human cancers (O'Brien et al., 2007; Moulder et al., 2008; Rai et al., 2008) because of its selective and specific down-regulation of Bcl-2 expression. Beside this effect, *in vitro* experiments with PLB indicated the attitude of G3139 to directly bind VDAC1 and to reduce channel conductance (Lai et al., 2006; Tan et al., 2007). Hence, exposure of isolated mitochondria to G3139 results in VDAC closure, accumulation of mitochondrial ROS and onset of cell death (Aggarwal et al., 2003; Tikunov et al., 2010).

Today, microRNAs (miRNAs) definitely represent an emerging tool in the treatment of cancer and neurodegenerative diseases. These molecules are short single-stranded RNAs of 21–24 nucleotides complementary to the 3′-end or, more rarely, to the 5′-end of mRNAs transcribed from target genes. Physiologically, microRNAs regulate gene expression at both transcriptional and post-translational level. Hence, because of their indispensable role in the control of numerous biological processes including cell cycle, cell growth, and apoptosis (Siomi and Siomi, 2010), considerable changes in their expression profiles have been associated with various diseases. In cancer, for instance, the simultaneous expression increase of miRNAs that act as oncogenes and decrease of others functioning like tumor-suppressors has been described (Di Leva et al., 2012). As the main actor in the Warburg metabolism, together with hexokinase, VDAC has been proposed as a target of miRNA modulation (Bargaje et al., 2012; Chaudhuri et al., 2016; Wang et al., 2016). Although there are still very few available data, the small non-coding RNA miR-7 would down-regulate the oncogene VDAC1 in hepatocarcinoma tissues, affecting cell proliferation and metastasis (Wang et al., 2016). Interestingly, Chaudhuri et al. described also a protective effect of miR-7 in cellular models of Parkinson disease, where it prevents depolarization of mitochondria by directly down-regulating VDAC (Chaudhuri et al., 2016) (Figure 3B, Group 4).

## Conclusions

This work focused on the significant contribution of VDAC in cancer and neurodegeneration, two types of diseases apparently different from each other. The detailed list reported above shows that all the pharmacological molecules, peptides and mRNAs described in the literature as targeted to VDAC are potentially effective in the therapeutic treatment of these pathologies. It is noticeable that numerous compounds able to induce apoptosis in cancer cells by modulating VDAC are already part of promising clinical trials. Many others, however, have been tested only *in vitro* or *in cellulo* and probably never will be applied in humans because of high toxicity or delivery difficulties. With regard to neurodegenerative diseases, the molecules that proved to suppress apoptosis and thus promote cell survival are still very few. This happens most likely because the molecular mechanisms underlying these disorders are still less characterized than those that define cancer. Although much remains to be to cover and uncover on the physiological role of VDAC in mitochondrial function and dysfunction, the overall data emphasize that targeted drugs and genetic approaches acting on VDAC represent encouraging strategies to treat a wide range of human diseases.

## Author contributions

AM and SR: collected the information and reference list for the manuscript. They also draw the figures. VDP: wrote the main part of the text and edited it.

### Conflict of interest statement

The authors declare that the research was conducted in the absence of any commercial or financial relationships that could be construed as a potential conflict of interest.
